# Correlation between serum leptin and bone mineral density in hemodialysis patients

**DOI:** 10.15171/jrip.2016.24

**Published:** 2016-07-22

**Authors:** Mahin Ghorban-Sabbagh, Fatemeh Nazemian, Massih Naghibi, Mohammad-Taghi Shakeri, Saeedeh Ahmadi-Simab, Reza Javidi-Dasht-Bayaz

**Affiliations:** ^1^Kidney Transplantation Complications Research Center, Montaseriyeh Organ Transplantation Hospital, Mashhad University of Medical Sciences, Mashhad, Iran; ^2^Nephrology Section, Department of Internal Medicine, Imam-Reza Hospital, Mashhad University of Medical Sciences, Mashhad, Iran; ^3^Department of Community Medicine and Public Health, Ghaem Hospital, Mashhad University of Medical Sciences, Mashhad, Iran; ^4^Cancer Research Center, Mashhad University of Medical Sciences, Mashhad, Iran; ^5^Faculty of Medicine, Mashhad University of Medical Sciences, Mashhad, Iran

**Keywords:** Hemodialysis, Leptin, Bone mineral density, Dual-energy x-ray absorptiometry

## Abstract

**Introduction:** For diagnosing of specific types of bone lesions in hemodialysis (HD) patients, it is necessary to conduct a bone biopsy as the gold standard method. However, it is an invasive procedure. While different markers have been suggested as alternative methods, none of them has been selected. The frequency of hip fractures is 80 fold in HD patients who have two-fold mortality as compared with general population.

**Objectives:** Recently, serum leptin has been suggested as a bone density marker. This study tries to confirm this proposal.

**Patients and Methods:** In this study about 104 HD patients (53.8% male and 46.2% female) were enrolled. The average age was 38.28±7.89 years. Serum leptin, bone alkaline phosphatase, intact parathyroid hormone (iPTH), 25(OH)D, calcium, phosphorus and bone mineral density (BMD) (at the femoral neck and lumbar spine, as measured by dual-energy x-ray absorptiometry [DXA]) were assessed.

**Results:** Analysis by polynomial regression revealed no correlation between BMD Z-score at two points and serum leptin level. According to the thresholds of 25 ng/mL and 18-24 ng/mL in some studies, we detected 25 ng/mL as the threshold in our patients. Under this threshold, the leptin effect on bone mass was negative, and above the threshold of 25 ng/mL, we found leptin had positive effect on bone mass.

**Conclusion:** In this investigation, we found, leptin has a bimodal effect on bone mass. Cortical bones assessment may be a better option for assessment.

**Implication for health policy/practice/research/medical education:**

In a study on 104 HD patients, we found, serum leptin has a bimodal effect on bone mass.

## Introduction

Chronic kidney disease-mineral and bone disorder (CKD-MBD) is a familiar term for clinicians and nephrologists since 2005. All hemodialysis (HD) patients have different percentages of bone involvement. Except “bone biopsy” until now, scientists have not found an exact non-invasive way for detecting the type and degree of bone involvement in this group of patients. Bone densitometry does not reliably predict fracture risk in patients with glomerular filtration rate (GFR) <45 mL/min/1.73 m^2^ and neither does it predict the type of renal osteodystrophy (KDIGO 2012) however, recent data support that dual-energy x-ray absorptiometry (DXA) can predict fracture risk in patients with CKD. Importantly, DXA remains an inexpensive and widely available technique that can easily be standardized across sites. Given this reliability, DXA is likely to be a good tool in longitudinal CKD research studies for the serial assessment of bone mineral density (BMD) in response to interventions (1). But, if we have more knowledge about what happens in the HD bones, leptin is produced predominantly in the adipose tissue. However, it is also expressed in a variety of other tissues, including placenta, ovaries, mammary epithelium, bone marrow, and lymphoid tissues. Leptin binds to leptin receptors (ObRs) located throughout the central nervous system and peripheral tissues, with at least six identified receptor isoforms (ObRa, ObRb, ObRc, ObRd, ObRe, and ObRf). The kidneys play a significant role in the plasma removal of leptin. Given its size (16000 Da), leptin is freely filtered by the glomerulus. However, while leptin is freely filtered, little or no leptin cleared by the kidneys appears in the urine. Renal processing of leptin appears to occur through renal tubular uptake and cellular degradation (1,2). Although not all patients with CKD have elevated serum leptin levels as reported in some studies, a significant elevation is noted when such levels are adequately corrected for body fat mass and age (2). The leptin that accumulates in patients with CKD is a bioactive and free form and not a protein-bound one, which is most prevalent in lean healthy controls.

In a study, in patients with moderate renal insufficiency (serum creatinine concentration of 2.5 mg/dL [221 µmol/L]) no renal clearance of leptin was observed. The exact cause of elevated serum leptin in CKD patients is not well understood. Various data suggesting that several factors may be involved, including loss of functioning renal mass, dialyzer membranes, dialysis modality, low erythropoietin levels, chronic inflammation and hyperinsulinemia. The results of some studies consistent with relatively high leptin levels caused weight loss in patients with end stage renal disease (3). However, another study found no correlation between serum leptin and weight change in patients with hyperleptinemia and end-stage renal disease (ESRD) (2). Besides being as energy hemostatic hormone, leptin has been shown to be also involved in gonadal maturation and in somatotropic and adrenocorticotropic functions regulating the immune system and body development. It is only recently that it has been found to be involved in bone metabolism as well. In vitro studies have shown that it promotes differentiation of mesenchymal stem cells to osteoblasts rather than adipocytes and inhibits osteoclastogenesis by increasing osteoprotegerin and decreasing RANK-ligand and synthesis (4). Furthermore, in vivo experiments of systemic leptin administration has proved to stimulate bone growth, to increase bone strength and to prevent ovariectomy induced bone loss (5). In contrast, its intracerebroventricular administration in wild or ob/ob leptin deficient mice resulted in bone loss (6). According to various papers about the relation between serum leptin and BMD published between 2007 and 2014, 18 papers showed no correlation between these two elements after adjusting correlation for the age, gender, or hormonal levels among others (7-25). In the remaining four papers, one paper showed a positive correlation among pre-pubertal girls (48 cases) (13). While another paper, showed that leptin is inversely associated to BMD in Brazilian obese adolescents (109 cases) (23). The other study was done on 52 Brazilian HD patients with the average age 57 and certified correlation between leptin and BMD with bone biopsy (25). Finally, the last one showed that leptin was positively related with the whole body and femoral BMD in postmenopausal nondiabetic elderly women (63 cases) (22). However, according to their limited cases, these results should examine in other studies. Moreover, nutritional and drug agents also can affect bone density. For example, long-term warfarin therapy in patients with valvular heart disease and prosthetic heart valves could significantly decrease bone density in lumbar spine region. Therefore, these factors also must be considered in all studies with bone density assessment (26).

## Objectives

I n this study we assessed repeatedly the correlation between leptin and BMD in our young and middle-aged HD patients. The majority of prior studies was done on elderly people who may suffer from senile osteoporosis; however, our patients were between 20-55 years old. Moreover, another priority in this study is the sample size of 104 patients. Additionally, the correlation of other laboratory parameters with leptin was also investigated.

## Patients and Methods

### Study population

This cross-sectional study was carried out between 2010 and 2012 in all HD centers in Mashhad, Iran. Around 115 patients between the ages 20 to 55, were included to this study, however 104 patients (56 males and 48 females) were eligible to include to the study. All of them were treated by conventional HD about 4 hours with bicarbonate base dialysate and polysulfone membrane, three-times a week and all had KT/V ≥1.2 in recent three months. Our patients had at least 6-month history of initiation of their HD. The exclusion criteria consisted of no history of malignancy, glucocorticoid consumption in recent one year ago, previous fracture (from initiation of HD) and previous parathyroidectomy (that can affect bones) or previous history of kidney transplantation.

### Assays

All samples were given by expert HD nurses after about 12 hours fasting. The serum obtained after centrifugation was stored in aliquots at -20°C before it was assayed. Body weight and height of all patients were recorded after HD session and body mass index (BMI) (kg/m^2^) was calculated. All tests were analyzed by enzyme-linked immunosorbent assay (ELISA) method by using German LDN kit for leptin (normal value;3.7-11.1 mean 7.4 ng/mL for female and 2-5.6 mean 3.8 ng/mL for male), and intact parathyroid hormone (iPTH) (normal value: 7.6-37 ρg/mL) and 25 OHVD and bone ALP (bALP) (normal value; 3.7-11.1 U/L) using German IDS kits of immunodiagnostic system kits.

### Dual X-ray absorptiometry

BMD of our patients was measured at femoral neck (as a mixed bone) and lumbar spine (as a cancellous bone) level using DPX-L densitometer (Lunar, Madison, Wis, USA). All BMD measurements were performed by the same experienced operator. BMD results were obtained in absolute values (g/m^2^) in T-scores and Z-scores. T-score is the number of standard deviations (SDs) from the mean BMD for young sex-matched normal controls (20-40 years old) and Z-score is the number of SDs from the mean BMD for age and sex-matched normal population, which allows the comparison of BMD between patients of different ages and genders. The reference values were obtained from Italian normal range.

### Ethical issues

The research followed the tenets of the Declaration of Helsinki. All participants gave their informed written consent to enter the study. Participation in this study was voluntary and patients were thus free to withdraw from the study at any time without having any effect on their treatment process. This study was approved by the ethic committee of Mashhad University of Medical Sciences.

### Statistical analysis

Total number of patients in our study were 104 HD patients, of whom 48 (46.2%) were female and 56 (53.8%) were male. As in our statistical analysis, the data are of the type normally distributed, they have been presented in the form mean± SD. Data were analyzed by SPSS software (version 16, SPSS Inc., Chicago, IL, USA). We used descriptive statistics, *t* test, Kolmogorov-Smirnov Z and correlation for data analysis. In our study, significance was defined when *P*< 0.05.

## Results

### Demographic and biochemical data

All our subjects were 104 HD patients, of whom 48 (46.2%) were female and 56 (53.8%) were male (Table 1). The demographic and biochemical data are shown in Table 1. Female patients were from 22 to 50 years old. Male patients’ ages ranged from 20 to 50 years old. Mean±SD of serum leptin, iPTH and bALP were 34.03 ± 22.62 (ng/mL), 206.51±174.89 (ρg/mL) and 74.41±73.62 (U/L), respectively.

**Table 1:** Demographic and biochemical markers of our patients (Mean± SD)

	**ALL**	**Female**	**Male**	***P*** ** value** ^1^
Age (y)	38.28 ± 7.89	38.52 ± 7.50	38.07 ± 8.27	NS^1^
P (mg/mL)	5.35 ± 1.84	5.22 ± 1.90	5.47 ± 1.79	NS^1^
Ca ‏(mg/mL‏)	8.78 ± 1.27	8.50 ± 1.26	9.03 ± 1.24	0.034
Leptin ‏(ng/mL)	22.62 ± 34.03	39.75 ± 42.38	8.07 ± 13.08	0.000
iPTH (pg/mL)	206.51 ± 174.89	222.42 ± 166.33	192.6 ± 182.41	NS^1^
Vitamin D (ng/mL)	17.04 ± 9.05	13.90 ± 9.15	19.7 ± 8.14	0.001
Duration (y)	4.85 ± 4.36	4.65 ± 4.23	5.03 ± 4.51	NS^1^
BMI (kg/m^2^)	22.83 ± 3.64	23.65 ± 3.72	22.13 ± 3.45	0.034
bALP (U/L)	74.41 ± 73.62	77.2 ± 75.03	72.04 ± 73.04	NS^1^
Femoral-Z	-1.13 ± 1.18	-1.21 ± 1.14	-1.04 ± 1.21	NS^1^
Femoral-T	-1.66 ± 1.27	-1.63 ± 1.27	-1.68 ± 1.28	NS^1^
Lumbar-Z	-0.85 ± 1.48	-0.77 ± 1.46	-0.93 ± 1.52	NS^1^
Lumbar-T	-1.30 ± 1.63	-0.92 ± 1.72	-1.68 ± 1.43	0.022

^1^Non-significant.

BMI in our female patients was significantly higher than males (23.65±3.72 kg/m^2^ versus 22.13±3.45 kg/m^2^, *P*=0.034). Leptin levels were significantly higher in females than males (39.75 versus 8.07; *P*<0.001). Around 30 patients had leptin levels of ≥25 ng/mL and 68 patients had leptin levels of <25 ng/mL.

In all patients, a positive significant correlation between leptin and BMI (*P*<0.001, *P*=0.017 and *P*<0.001, respectively) was detected.

There are no significant differences between two genders among some variables such as age, phosphor, intact PTH and duration on HD. The calcium level was significantly lower in females than males (8.5±1.26 versus 9.03±1.24). The leptin level was significantly higher in females than males (42.38±39.75 versus 13.08±8.07ng/mL) (*P*=0.0001). Vitamin D level was significantly lower in females than males (13.90±9.15 versus 19.70±8.14 ng/mL) (*P*=0.001). Women had significantly higher BMI than men (23.65±3.72 kg/m^2^ versus 22.13±3.45 kg/m^2^) (*P*=0.026). Correlation between serum leptin level and the other variables in two genders are shown in Table 2.

**Table 2:** Correlation between leptin and variables

	**All**		**Female**		**Male**	
	**Correlation**	***P *** **value**	**Correlation**	***P *** **value** ^1^	**Correlation**	***P *** **value**
BMI (kg/m^2^)	0.43	0.000^*^	0.353	0.017^*^	0.637	0.000^*^
P (mg/dL)	0.037	0.722	0.084	0.582	0.098	0.489
Ca (mg/dL)	-0.122	0.234	-0.024	0.874	-0.035	0.806
Femoral-Z	0.089	0.403	0.252	0.099	-0.063	0.677
Femoral-T	0.241	0.022^*^	o.370	.013^*^	0.112	0.460
Lumbar -Z	0.119	0.269	0.255	0.095	-0.258	0.091
Lumbar-T	0.236	0.027^*^	0.203	0.185	0.003	0.982
Duration on HD (y)	-0.192	0.059	-0.283	0.060	-0.054	0.706
bALP (U/L)	-0.061	0.548	-0.075	0.626	-0.17	0.223
Age (y)	-0.082	0.421	-0.199	0.191	0.001	0.992
iPTH (pg/mL)	-0.026	0.799	-0.083	0.587	-0.138	0.325
Vitamin D (ng/mL)	-0.311	0.002^*^	-0.217	0.157	-0.200	0.154

* P value < 0.05

Additionally, an inverse significant correlation of serum leptin and vitamin D level in all patients was detected (r=-0.311, *P*=0.002). Correlation between leptin and bALP was negative in both males (r=-0.17, *P*=0.223) and females (r=-0.075, *P*=0.626), and in all groups (r=-0.061, *P*=0.548), however, none of them was significant. The relation between duration of HD and leptin levels also was negative but not significant (r=-0.192, *P*=0.059). There were positive correlations between serum leptin and lumbar T-score (r=0.236, *P*=0.027) and femoral T-score in all patients (r=0.241, *P*=0.022) and also in females (r=0.370, *P*=0.013). We did not find any correlation between other BMD parameters and leptin level (*P*>0.05). Lumbar densities in all patients were significantly better than femoral densities (-0.85 ± 1.48 in lumbar region and -1.09 ± 1.17 g/cm^2^ in femoral neck region) (*P*<0.05).

**Figure 1:**
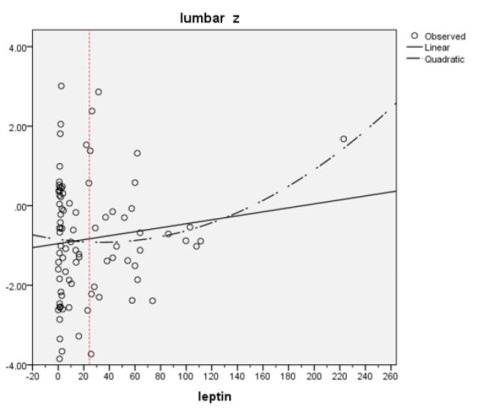
Correlation between lumbar bone density and peripheral serum leptin (ng/mL) in hemodialysis patients. Polynominal regression analysis with all leptin values: y=6.59×10-5x2– 4×10-3x – 0.846, n=98, r=0.029, *P*=0.282; simple regression analysis for leptin>24.41 ng/mL: y=0.007x – 1.099, n=30, r=0.033, *P*=0.335.

**Figure 2:**
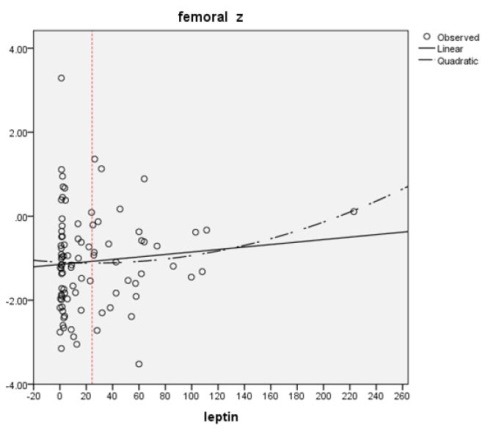
Correlation between femoral neck bone density and peripheral serum leptin (ng/mL) in hemodialysis patients. Polynomial regression analysis with all leptin values: y=3.2×10-5x2–2×10-3x–1.096, n= 98, r=0.014, P=0.55; simple regression analysis for leptin>24.41 ng/mL: y=0.003x – 1.133, n=30, r=0.011, P=0.575.

Figures 1 and 2 show the simple regression analysis between z-scores of lumbar and femoral neck regions with leptin levels above and under 25 ng/mL. We observed a correlation between two groups of relations, negative to positive values (r: -0.053 in femoral and -0.024 at lumbar region in leptin values under 25 ng/mL and 0.107 and 0.182 in leptin values above 25 ng/mL). The *P* values at femoral neck and lumbar spine regions were 0.575 and 0.335 in values ≥25 ng/mL and 0.685 and 0.857 in values <25 ng/mL that were not significant (Tables 3 and 4).

**Table 3:** Correlation between leptin ≥25 and BMD in femoral neck & lumbar spine region (Z-score)

		**Femoral-Z**	**Lumbar-Z**
Leptin (ng/mL)	Correlation	0.107	0.182
	*P* value	0.575	0.335

**Table 4:** Correlation between leptin <25 and BMD in femoral neck & lumbar spine region (Z-score)

		**Femoral-Z**	**Lumbar-Z**
Leptin (ng/mL)	Correlation	-0.053	-0.024
	*P* value	0.685	0.857

## Discussion

Our data on total serum leptin in our patients confirmed the gender dimorphism. The leptin levels in females were five times higher than males (42.38±39.75 versus 13.08±8.07). Other studies also showed this priority in females (27-31). Considine et al pointed out that women and men with equivalent percentages of body fat had comparable levels of serum leptin. We did not have this type of comparison in our cases (32). In the study by Ahmadi et al, leptin levels were higher in males than in females (24). In healthy general population (BMI<25) leptin level in women was measured to be more than that of men (women; 6.9±4.3 versus men; 5.6±2.2) (*P*≤0.05) (33).

BMI in our female patients was significantly higher than males (23.656±3.72 versus 22.136±3.45; *P*=0.034). In assessment of correlation between leptin levels and BMI, in all patients, a positive and significant correlation between them was seen (*P*<0.001, *P*=0.017 and *P*<0.001, respectively). Except in the study by Ahmadi et al (24), which they did not find any correlation between leptin and BMI, the majority of other studies find a significant correlation between them (15,27-31).

In assessment of correlation between serum leptin level and BMD, various studies were published (24,27,28,34). In all studies that were done at femoral neck and lumbar spine, any correlation between leptin level and BMD was detected (24,27,28). However, this correlation at different sites of radius bone (compact bone) was found to be significant (27,34). In this study we investigated this relationship in our patients. We selected young and middle-aged patients (20-55 years) and thus we have tried to limit the effect of the senile osteoporosis as a confounder agent. However, we did not find any significant correlation between leptin level and BMD in our patients. In this study we used Z-score as a main indicator of BMD in our patients in order to access to more real information instead of T-score, and also from same definitions that used in general population. Polynomial regression analysis showed that the correlation curve between the BMD Z-score and serum leptin had a flat U-shape with an inflexion point of 25 ng/mL of serum leptin. This corresponds with saturation threshold previously expressed by Caro et al (33) and Ghazali et al (27) who suggested a threshold (17-24 ng/mL) in their patients. We also detected approximately the same threshold in our patients. Under this threshold the relationship (r) between leptin and bone mass was negative (r=-0.053, -0.024 for femoral neck and lumbar region respectively), and above that this relation (r) was positive (r=0.107, 0.182 for femoral neck and lumbar region respectively). As mentioned earlier, *P* values at femoral and lumbar region were 0.575 and 0.335 in values ≥25 ng/mL of leptin and 0.685 and 0.857 in values <25 ng/mL of leptin that were not significant. In Ghazali et al (27) study in patients with serum leptin above the curve inflexion point, they showed significant positive correlations at both radius sites (r=0.53, *P*=0.03 at midshaft radius and r=0.50, *P*=0.04 at ultradistal radius). Ghazali et al also did not find any significant correlation at femoral neck level (27).

An interesting point is that in all investigations performed until now, bone biopsy from iliac crest has been as gold standard test for detection of bone changes. As mentioned earlier, while, in the majority of studies done until now, no significant relations between lumbar spine density and leptin level has been detected, all bone biopsies are done on this region. This is done in spite of the fact that assuming any significant and meaningful relation in this context may seem irrational.

Trabecular bones in our body have different roles. For example, metabolic and weight bearing roles. They are very accessible and walkaway in view of their structures. For these reasons and several other factors that we do not know about them. It seems that they are not good candidates for leptin effect assessments. However, cortical bones seem to be less under metabolic and weight bearing effects in our body and therefore they can be better representor of pure bone changes in bone diseases. Jamal et al in 2007 showed that cortical bones (ultra-distal radius) had significant differences in bone density between two groups of dialysis patients with and without fractures (35). In Ghazali et al (27) and Coen et al (29) studies also, a significant relation between bone density at mid-shaft and ultra-distal of radius and leptin level was detected.

## Conclusion

We believe that leptin has a bimodal effect on bone mass with a threshold as previously mentioned in the studies of Caro et al (33) and Ghazali et al (27). Maybe the assessment of this effect has not been shown correctly by iliac crest bone biopsy as a trabecular bone. Cortical bones assessment may be better candidate.

## Limitations of the study

The limitation of our study was small sample size of voluntary patients. Therefore, a multicenter trial may result in a better revaluation of this aspect of HD patients.

## Acknowledgements

The authors wish to thank HD wards of Imam-Reza and Montaseriyeh hospitals of Mashhad University of Medical Sciences, Mashhad, Iran, to support this study and all the colleagues and nurses who participated in the data collecting process.

## Authors’ contribution

All authors contributed to design of the research. FN supervised the research. MGS conducted the research and prepared the manuscript. RJDB helped in collection and analyzing the data. MN, MTS and SAS commented, gave their advice and helped during the research. All authors read, revised and approved the final manuscript.

## Conflicts of interest

The authors declare that they have no conflicting interest.

## Ethical considerations

Ethical issues (including plagiarism, data fabrication, double publication) have been completely observed by authors.

## Funding/Support

This manuscript is issued from the nephrology fellowship thesis of Mahin Ghorban-Sabbagh and was supported financially by Mashhad University of Medical Sciences, Mashhad, Iran (Grant# t-2436).
